# Perspectives from research and practice: A survey on external load monitoring and bone in sport

**DOI:** 10.3389/fspor.2023.1150052

**Published:** 2023-04-25

**Authors:** Reece Scott, Ruth James, Cleveland T. Barnett, Craig Sale, Ian Varley

**Affiliations:** ^1^ Musculoskeletal, Physical Activity and Metabolic Health Research Group, Sport, Health and Performance Enhancement Research Centre, School of Science and Technology, Nottingham Trent University, Nottingham, United Kingdom; ^2^ Institute of Sport, Department of Sport and Exercise Sciences, Manchester Metropolitan University, Manchester, United Kingdom

**Keywords:** external load, elite sport, bone load, qualitative, measuring devices

## Abstract

**Introduction:**

There is limited information regarding the association between external load and estimated bone load in sport, which may be important due to the influence exercise can have on bone accrual and injury risk. The aim of this study was to identify external load measuring tools used by support staff to estimate bone load and assess if these methodologies were supported in research.

**Methods:**

A survey was comprised of 19 multiple choice questions and the option to elaborate on if/how they monitor external load and if/how they used them to estimate bone load. A narrative review was performed to assess how external load is associated to bone in research.

**Results:**

Participants were required to be working as support staff in applied sport. Support staff (*n* = 71) were recruited worldwide with the majority (85%) working with professional elite athletes. 92% of support staff monitored external load in their organisation, but only 28% used it to estimate bone load.

**Discussion:**

GPS is the most commonly used method to estimate bone load, but there is a lack of research assessing GPS metrics with bone load. Accelerometry and force plates were among the most prevalent methods used to assess external load, but a lack of bone specific measurements were reported by support staff. Further research exploring how external load relates to bone is needed as there is no consensus on which method of external load is best to estimate bone load in an applied setting.

## Introduction

Physical loading can be separated into two categories; internal load and external load (1). Internal load is the biological stress imposed upon an individual, such as heart rate or blood lactate (2), whereas external load can be described as the work completed (e.g., acceleration or force) independent of the internal characteristics (3). Biomechanical external load has an important relationship with the mechanical stresses imposed on the musculoskeletal system (4). Monitoring external load is important in sport as it provides objective data on physical attributes in response to prescribed training (5), as well as being used to optimise performance (6, 7). High intensity external loading has been associated with an increase in injury risk of up to 270% in rugby league (8) and football (9) and, as such, monitoring external load in applied settings has increased in popularity to try to mitigate against injury (10, 11). Understanding methods to measure external load in relation to bone allows support staff to gain the knowledge of how external load can be associated to bone characteristics and its applicability within an applied setting. Subjective methods (e.g., questionnaires or rating of perceived exertion) are often used to monitor load in athletes, but these metrics lack reliability and validity in comparison to objective measures as these are dependent upon the athlete's perception (12). Although invasive methods (e.g., bone mounted strain gauges and bone staples) studying bone responses are insightful, they are restricted in their applicability due to their invasiveness (13). Blood biomarkers such as N-terminal propeptide of type I procollagen and C-terminal telopeptide of type I collagen are less invasive methods of understanding the response in bone, however these do not quantify bone load but rather provide information relating to the bone (re)modelling response at the whole body level (14). Metrics derived from applied technologies (e.g., global positioning systems, GPS; inertial measurement units, IMU; force plates; motion capture) do, however, have the potential to be associated with bone characteristics. Associations between bone characteristics and physical performance (e.g., high-speed distance associated with bone mass, trabecular density and cortical density, and peak speed associated with bone mass, cortical density and thickness) indicate bone is influenced by exercise intensity. For example, acceleration and total distance derived from GPS were positively correlated with bone mineral content (BMC) and tibial strength in footballers (15). Further studies have attempted to understand the relationship between physical activity and bone, although these have been performed in nonathletic populations (16–18) or associated to injury (19) rather than bone structural characteristics.

Associations between data derived from external load measuring devices and bone are not well established in an applied setting. Accelerometery data have been linked to changes in bone with vigorous physical activity being associated to higher bone mineral density (BMD) and BMC (16). Higher ground reaction forces (GRF) are linked to greater osteogenic loading (20) with GRF intensity thought to be a better predictor of BMD and skeletal adaptations than loading volume (21). IMU's are a novel approach to monitoring bone stimulus in the field utilising site-specific segmental acceleration as opposed to whole-body loading that GPS devices measure (22–24). IMeasureU (Auckland, New Zealand) offer a bone stimulus metric that combines the number of loads and magnitude of loads to predict the stimulus response of bone (22), but these claims are unsubstantiated. GPS is a more popular method to monitor external load in the field and are often used to inform rehabilitation strategies for soft tissue injuries (10) and to determine outcomes considered pertinent to performance (6), although the use of GPS metrics and their relationship to bone adaptation is unclear. Acceleration derived metrics from IMU's and GPS are often used for quantifying external load (25), yet it is not elucidated whether these measures can be associated to bone characteristics. Therefore, the efficacy of monitoring external load to gauge bone stimulus in an applied environment is not well known.

**Figure 1:**
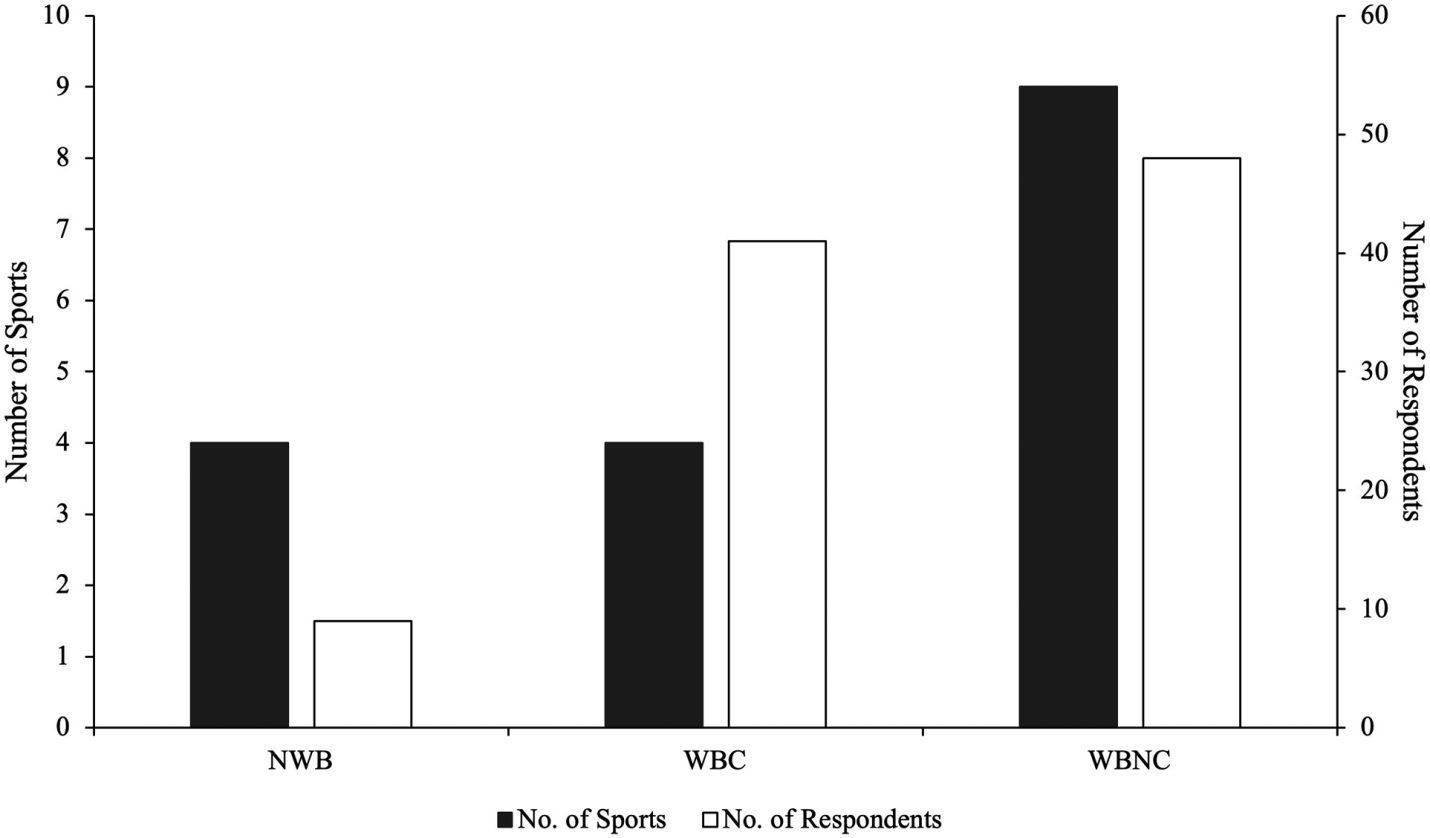
Number of survey respondents alongside number of sports classified into sporting conditions. Sporting conditions are classified as; non weight-bearing sports (NWB) include cycling, swimming, canoeing and rowing; weight-bearing contact sport (WBC) include Football/Soccer, Rugby, Judo and American football; weight-bearing non-contact sport (WBNC) include Cricket, Athletics, Basketball, Volleyball, Field Hockey, Baseball, Triathlon, Dance and Squash.

Bone stress injuries (BSI) are often associated with alterations within training programmes (26) and, as such, an ability to monitor external load upon the bone accurately and reliably offers the potential to support risk mitigation strategies for BSI's by helping to ensure that athletes are not exposed to sudden excessive loading cycles. It is argued that external load monitoring can be used to manage bone load and reduce the incidence of BSI, as prompt increases in load are prominent in their pathophysiology (27). Whilst this might be the case, it remains unknown to what extent athlete support staff estimate bone load, and, if they do, what methods and metrics they use to do so, given that there is no consensus on the optimal method for monitoring changes in bone.

Therefore, the aims of this study were two-fold; (1) to identify the methods used to monitor external load and ascertain if these methods are used to estimate bone load by surveying support staff, and (2) to assess the measurement tools used to estimate bone load through a narrative review.

## Materials and methods

### Participants

Support staff (*n* = 71) from sports clubs or national governing bodies (Figure 1) were recruited worldwide (UK *n* = 48, 67%; Rest of Europe *n* = 7, 10%; North America *n* = 7, 10%; Australia *n* = 5, 7%; Africa *n* = 2, 3%; Asia *n* = 1, 1%; South America *n* = 1, 1%) *via* email or word of mouth. The role occupied by those surveyed included: Strength and Conditioning Coach (*n* = 29), Sports Scientist (*n* = 22), Physiotherapist (*n* = 13), Coach (*n* = 2), Physiologist (*n* = 1), Sports Therapist (*n* = 1), Athletic Trainer (*n* = 1), Researcher (*n* = 1) and Nutritionist (*n* = 1).

### Procedures

Participants were asked to provide informed consent and complete a survey related to external load monitoring and bone in sport between July 2020 and August 2020. The internet-based survey platform (Jisc, Bristol, UK) was used, with the survey being completed anonymously. It comprised of 19 multiple choice questions relating to external load monitoring in sport. Respondents were able to elaborate on their answer with the “Other” option if they wished to do so.

Participants met the inclusion criteria if their role involved working in a support staff capacity in an applied sporting environment. Prior to taking part in the study, each participant provided informed consent. Ethical approval was granted by the Non-Invasive Human Ethics Committee from Nottingham Trent university (126V2). The data that support the findings of this study are openly available in figshare at 10.6084/m9.figshare.21764135 or available upon request.

The survey (Appendix A) divided the topic of “external load monitoring” into two sections; (a) if/How external load is monitored and, (b) what methods/metrics are used to estimate bone load. Multiple choice and free text options were provided on the common methods identified within research for sports performance and external load quantification. Frequency based descriptive analyses were performed upon fully completed surveys.

Alongside the survey, a narrative review was performed using PubMed as a database to assess how external load is associated to bone. Google Scholar was used as a complimentary database. Articles were sourced and reviewed by two of the authors (RS, IV). The search strategy used the keywords “bone load”, “external load”, “non-invasive bone load” and “bone and exercise”. Articles were included if they met the following:

• The methodology presented in the study was non-invasive (meaning the research was performed in an applied environment and not intrusive for participants)
• Human trials only
• The loading metric used had bone health, load or injury as outcome variables.
• Fully published, peer reviewed articles

Various metrics were reported within the studies, although the methodologies adopted were consistent between each study.

## Results

### Current use of external load monitoring in sport

The majority of support staff reported monitoring external load with their athletes (92%). For the 8% that did not monitor external load, this was primarily due to a lack of equipment (83%). The most common methods used by support staff were GPS, force plates, IMU and motion capture. Only 28% of support staff used these methods to estimate bone load, however, with 40% stating the main barrier was a lack of knowledge (Table 1).

**Table 1:** Support staff response to using external load monitoring in sport.

	Yes, *n* (%)	No, *n* (%)	Unsure, *n* (%)		
**Does your club/organisation monitor external load in your athletes? (*n* = 71)**	65 (92)	6 (9)	0 (0)		
*What is the primary reason you don’t monitor external load?* (***n* =** 6)	Lack of Time: 0 (0)	Lack of equipment: 4 (67)	Lack of knowledge: 0 (0)	Don’t feel it is needed: 1 (17)	Other: 1 (17)
**What systems do you use to monitor external load? (*n* = 65)**	GPS: 55 (85)	IMU: 11 (17)	Force Plates: 31 (48)	Motion capture: 10 (15)	Other: 16 (25)
**Do you use any of the external load metrics attained to estimate load on bone? (*n* = 65)**	18 (28)	43 (66)	4 (6)		
*What is the primary reason you don’t relate external load to bone?* (***n* =** 43)	Lack of Time: 6 (14)	Lack of equipment: 7 (16)	Lack of knowledge: 17 (40)	Don’t feel it is needed: 7 (16)	Other: 6 (14)

### Methods to estimate bone load

GPS was the most common method for monitoring external load (*n* = 55, 85%) and the most commonly used to estimate bone load (*n* = 11, 50%). The use of GPS to inform on bone related outcomes (21%–38%) was not, however, as prevalent as the use of IMU (50%–100%) or motion capture (40%–100%) for this purpose. Force plates were reportedly well utilised to monitor external load, but the least prevalent in relation to bone related outcomes (12%; Table 2).

**Table 2:** Support staff responses to the metrics used when measuring external load and the metrics used in relation to bone.

Method Metric	Respondents who measure external load *n* (%)	Respondents who measure external load to estimate bone load, *n* (%)	Prevalence of use to estimate bone load
**GPS**			
PlayerLoad	29 (45)	11 (50)	38%
Total distance	48 (74)	10 (46)	21%
High speed distance	47 (72)	10 (46)	21%
**IMU**			
Impact load	9 (14)	5 (23)	56%
Step count	3 (5)	3 (14)	100%
PPA	6 (9)	3 (14)	50%
**Motion capture**			
Torque	3 (5)	2 (9)	67%
Moment	5 (8)	2 (9)	40%
Stiffness	3 (5)	3 (14)	100%
**Force Plates**			
Peak ground reaction force	25 (39)	3 (14)	12%
RFD	26 (40)	3 (14)	12%
Impulse	17 (26)	2 (9)	12%
**Other**	19 (29)	7 (32)	37%

A total of 16 articles were included in the narrative review (GPS *n* = 1; Force plates *n* = 10; IMU *n* = 1; Motion capture *n* = 4) (Table 3).

**Table 3:** Articles included in narrative review (*n* = 16).

Measurement tool	Study (reference)	Experimental model	Main results
GPS (*n* = 1)	Varley et al. (15)	GPS training load across a football season × 3 time points. DXA and pQCT scans × 4 time points.	Correlations between training load variables and BMC and tibial strength.
Force plates (*n* = 10)	Jämsä et al. (18)	Postmenopausal women. 12 month high impact exercise intervention. 3× a week. GRF and accelerometer. DXA scan.	BMD change at proximal femur correlated with accelerations exceeding 3.6 g.
	Kohrt et al. (20)	Healthy older women. 9 month intervention, GRF and JRF exercise group. DXA scan.	Increase in whole body BMD for both GRF and JRF groups. Femoral neck BMD increase in GRF group.
	Rogers et al. (21)	Physically active middle aged men. Bone loading scores based off GRF exercise. DXA scan.	Bone loading during young adulthood was a predictor of BMD. GRF good predictor for increased BMD.
	Bailey et al. (37)	Premenopausal women. 50× hops 2×, 4× or 7× a week intervention for 6 months. GRF measures. DXA scan.	Femoral neck BMD significantly higher in 7 days a week group. BMC increased at femoral neck in 7 day group.
	Matijevich et al. (39)	Young healthy subjects. Treadmill run on range of slopes (−9 to 9 degrees) and speeds (2.6–4.0 m/s). Vicon motion capture and GRF. Lower extremity marker system. Model of tibial load.	Ankle force indicative of tibial bone load. GRF metrics not strongly correlated with increases in tibial bone load.
	Allison et al. (41)	Older men. 50× hops, 7 days a week intervention for 12 months. GRF measures. DXA scan.	BMD and BMC increased in the exercise leg and decreased in the control leg. Cross-sectional moment of inertia increased in exercise leg.
	Rantalainen et al. (45)	Healthy young men. Max GRF measured during bilateral jumping. Muscle torque measured with dynamometer. pQCT tibial scan.	GRF and eccentric torque positively correlated with tibial bone strength.
	Rantalainen et al. (46)	Premenopausal and postmenopausal women. CMJ performed on force plates. pQCT tibial scan.	Premenopausal group had higher bending and compressive bone strength. Higher peak GRF and impulse in premenopausal group.
	Wu et al. (51)	Rhythmic gymnasts. Muscle strength measured from IKD. GRF measured. DXA scan.	BMD higher in take-off leg and landing leg. Force significantly higher in take-off than landing leg.
	Rantalainen et al. (53)	Young male students. Bilateral jumping until exhaustion. GRF measured. Blood biomarkers measured.	Maximal GRF and P1NP marker were positively associated. Negative correlation between maximal GRF and CTX form pre and 2 days post intervention.
IMU (*n* = 1)	Besier. (56)	Bone stimulus metric created from number of cycles and peak strain.	No experimental data.
Motion capture (*n* = 4)	Milner et al. (19)	Habitual runners. Treadmill run at 3.7 m/s. Vicon motion capture. Lower extremity marker system. Tibial x-ray.	Greater vertical loading rate, impact peak, peak tibial shock and knee joint stiffness in tibial stress fracture group compared to controls.
	Laughton et al. (59)	Rear foot and forefoot strike runners. Running with and w/o orthotic devices. Accelerometer, GRF and motion capture. Model of lower limbs.	Positive correlations between peak positive tibial acceleration and anteroposterior GRF load rate. Forefoot strikers experience greater tibial shock.
	Choi et al. (60)	Older population. Barefoot walking over 9 m. Motion analysis and GRF. DXA of femoral neck.	Maximum hip power and BMD positive correlation in trochanter. Hip power-time integral positive correlation with femur.
	El Deeb et al. (61)	Postmenopausal women. 10 m walking gait trails. Qualisys motion system and GRF. Whole body marker system. DXA scan at femoral neck.	Low BMD associated with hip and trunk moments. Less power generated in hip with low BMD.

## Discussion

### Main survey findings

The current study aimed to identify the methods used to monitor external load in an applied environment and ascertain if these methods were used to estimate bone load. The secondary aim of this study was to perform a narrative review to assess if any external load methods give a reflection of bone load in the literature. The key findings of the study show external load is widely monitored by support staff, primarily using GPS, force plates, IMU and motion capture (Table 1), but fewer use it to provide insight on how bone responds to exercise and training. GPS was the most often used measurement tool related bone (Table 2). Although these methods have been validated and shown to be reliable for measuring performance related variables (28, 29), the validity of associating them with changes in bone is not well established (39).

### GPS and bone

GPS was the most common method of monitoring external load used by support staff (Table 2), likely due to its capacity for real time data interpretation (30). As GPS technology has developed, micro inertial sensors (triaxial accelerometers, magnetometers, gyroscopes) have been integrated into the devices, providing support staff with a wide range of metrics to indicate external load and undertake activity profiling (31). The use of GPS has been shown to offer an accurate and reliable method to quantify the habitual movement of athletes (4), although support staff should be cautious when measuring maximal accelerations as the sampling rate of some commercially available devices used in research (∼1 Hz; GPSports, Catapult innovations) may result in missed data over a short period of time (29). Furthermore, comparing high intensity running between GPS devices may be unreliable, as differences have been shown between manufacturers (29). Therefore, high intensity metrics (i.e., high intensity running) may not be reliable between devices as a result of high coefficient of variation (32.4%) when analysing high intensity movements.

Although GPS is used to monitor physiological markers relative to performance (31), less is known about how the metrics can monitor bone. This is despite our findings that total distance and high-speed distance are commonly used to estimate the load placed upon bone by support staff (46%; Table 2), where they may assume that greater distances covered, and higher peak speeds achieved result in greater bone loading. However, bone cells desensitise to repetitive, unidirectional loading (27, 32), meaning that total distance might be a less informative metric when it comes to determining the bone stimulus. This has been reported in rats where excessive high intensity loading (14,000 loading cycles on each limb per day) did not have beneficial or detrimental effects on bone (33). There are findings in humans that are similarly suggestive (27), with low intensity running shown to half the likelihood of BSI in comparison to high intensity running at an equivalent distance. Tibial strength and BMC were positively correlated to GPS training load metrics, such as acceleration, deceleration and total distance, in professional male footballers across a season (15). This suggests dynamic, high loading movements are important for creating an osteogenic response within bone, which may be monitored using GPS. The effect sizes in this study (15) were, however, moderate to low, suggesting that the practical use of GPS to estimate bone load is yet to be established. This may be why proprietary measures are used in applied settings to assess performance.

Data are available on PlayerLoad (commercially used metric to estimate workload completed in a given period) and distance covered in relation to sports performance and fatigue (3), but there is no robust scientific evidence to suggest that these metrics can be used to estimate bone load. Despite this, our findings show that PlayerLoad is the most prevalent metric (50%; *n* = 11 of support staff surveyed) used to estimate bone load. Strong correlations have been shown between PlayerLoad and the rate of pulmonary oxygen uptake and heart rate within participants (.92–.98), although a mixture of positive and negative moderate correlations were shown (−.38 to .33) between participants (28). Therefore, comparing PlayerLoad between athletes may not be reliable due to the variability of the measurement. The variability between participants suggests external differences in PlayerLoad may not reflect differences of internal load. Another issue with the reproducibility of PlayerLoad results from the ambiguity surrounding the measurement (34). This ambiguity stems from the inconsistent definitions presented within the literature surrounding the metric, resulting in a lack of clarity between studies (35, 36). Some research defines the variable as a vector magnitude representing the sum of accelerations from each direction (35), whereas others define it as being the instantaneous change in rates of resultant accelerations over time representing the acceleration load for an activity (36). Thus, this limits the application of PlayerLoad as a tool for monitoring external load as well as using this as a metric to estimate bone load. Research needs to clearly present how PlayerLoad is being calculated in order to offer a standardised and reproducible metric for support staff to understand.

A clear limitation of GPS devices is the sensor location, which is commonly positioned between the shoulder blades. The range of reliability statistics of positioning a GPS at the scapulae (ICC: .60–.93, CV: 4.%6–18.2%) compared to the centre of mass (ICC: .65–.97, CV: 3.6%–14.7%) shows a moderate to high test-retest reliability for both locations (28). However, wearing the device at the scapulae can underestimate some metrics (i.e., PlayerLoad) due to the lack of sensitivity to subtle movements during high-speed running (28). Stress related bone injuries occur predominantly in the lower limbs (26) and, therefore, measuring load at the scapula may not be a valid method for understanding the forces experienced in the lower limbs due to the greater distance from ground contact. Associations between GPS derived metrics and bone characteristics are not well established. The 21%–38% of support staff who relate GPS metrics to bone should do so with caution as there is no clear evidence on how the derived metrics can be used in relation to bone outcomes. This dearth of robust scientific data should be addressed by researchers as soon as possible.

### Force plates and bone

Force plates were reported to be the second most utilised method for measuring external load within the current survey (*n* = 31, 48%). Force plates are mechanical sensing systems that measure GRF's when contact is made with an external force (i.e., an athlete) and can be used to gather kinetic data. Support staff often use these devices within a gym environment to measure the effects of training programmes. Despite only ∼14% of support staff using GRF in relation to bone, researchers have shown associations between GRF and bone load/mechanical stimuli (20, 21).

The femoral neck, trochanter and Ward's triangle have shown an increase in BMD when daily impacts and accelerations are more frequent over a 12-month exercise intervention (18). Increases in BMD at the femoral neck have also been shown with 7 day a week hopping, offering a greater increase to BMD than hopping for 2 days or 4 days, with exposure to GRF's being the only difference between groups (37). This could be important for offering guidelines on exercise induced bone load without increasing exposure to injury. Internal forces acting upon bone are higher than surrogate measures, such as GRF, but the accessibility of GRF's means they can be used as a guide for the intensity of internal bone loading during defined exercises (41). Weight bearing exercise (walking, jogging and stair climbing) has been shown to increase BMD more than resistance training (20). This was associated with having a higher loading force and faster loading rate when performing weight bearing activity. Although this demonstrates that BMD may increase with loading, the range of load and how varying loads influence bone characteristics have not been investigated. Exercise intensity may be more important than exercise frequency for bone accrual (21). Classifying bone load into categories: 0 (GRF 1 × bodyweight); 1 (GRF between 1 and 2 × bodyweight); 2 (GRF between 2 and 4 × bodyweight) and 3 (GRF > 4 × bodyweight) has shown greater bone load (category 3) has a positive linear relationship with whole-body BMD and a positive effect on skeletal health in later life (21). The applicability of this scoring system in relation to bone load is questioned due to two points; (1) the lack of validity surrounding the biomechanical GRF (38) and (2) the retrospective measure of physical activity across the lifespan. As far as the current authors are aware there is no validation for the GRF scores relevant to bone loading and, therefore, although it might be a solid concept, it cannot be truly classified as being a bone loading metric. Furthermore, as is highlighted in the study (21), the recall of physical activity across the lifespan from a middle-aged population is likely to have significant error, which will have affected the bone loading metric. The lack of assessment for diet, hormonal status and use of medication across adolescence and adulthood, which can all effect bone, also contribute to the lack of validity of comparing bone load to bone status. Using GRF in this format has potential to inform on intervention strategies in an applicable and simple technique but further validation of GRF in relation to bone load needs to be examined for it to be attempted as a predictor of skeletal adaptation.

Contrastingly, GRF's have been shown to be misleading for monitoring load in relation to bone. Research has shown that GRF does not correlate with tibial load as it does not account for muscular contraction (39). GRF's are not representative of internal multi-axial stress and may have little influence on the mechanical behaviour of bone relative to loading magnitude (40). Although muscular force applies the highest load on bone, GRF's still account for ∼30% of bone load (39), thus it is argued that it can still be used as a guide to the relative intensity of internal bone load during hopping exercises (41). Overall, GRF is associated to changes in bone within exercise interventions. The studies are often performed on nonathletic populations (i.e., post-menopausal women or adolescents) as opposed to athletes, so there are limited data that can be applied to the active athlete.

The ability to create a high force rapidly during muscular contractions may be a relevant measurement to inform on bone adaptations due to the functional link between muscle and –bone, in which both biological structures directly influence one another (42, 43). Rate of Force Development (RFD) is reportedly used by ∼14% of staff (*n* = 3) who use force plates to estimate bone load. The incorporation of time-based analysis makes the measure more indicative of neuromuscular performance, as opposed to peak force which may be more indicative of movement strategies (44). Neuromuscular performance has been associated with bone strength (45) as tibial strength was higher in those that produce greater eccentric torque, as well as predicting bone strength in pre- and postmenopausal women (46). Muscular forces impose a large load on bone demonstrated by the associations shown between bone growth and muscle growth (47). Therefore, using RFD alongside other GRF derived metrics may be advantageous to assess adaptations in bone (48), as muscular forces influence bone characteristics. There is evidence of muscle disuse and reuse causing changes to bone mass (49) and positive correlations are frequently reported between muscle mass and bone mass (50). The impracticality of measuring RFD in an applied setting is a possible drawback. Force plates are challenging to employ within sports that have spontaneous movement patterns, thus somewhat limiting the applied use of the method.

Impulse is the product of a resultant force and the duration of this force. Impulse was used by ∼9% (*n* = 2) of support staff who use external load monitoring in relation to bone. Impulse incorporates neuromuscular performance and body mass and has been shown to have a strong linear association with maximal power. Neuromuscular performance, represented by impulse, has been related to skeletal robusticity (skeletal strength relative to body size) through a regression model (46) with a 1% improvement in impulse associating to a 0.5% increase in skeletal robusticity. This is proposed as an alternative to using body mass alone as a predictor of skeletal robusticity, thus longitudinal measurements (i.e., CoM accelerations) may offer better estimates of bone load. The sample comparison of skeletal robusticity between active premenopausal women and inactive postmenopausal women favours the active population in bone strength (46). Therefore, to understand the effects of neuromuscular performance to estimate bone load it would make sense to examine healthy athletes alongside a less active population of a similar age. Greater impulse during a leaping take-off in rhythmic gymnasts was associated with higher BMD at the femur and bone strength at the knee extensors/flexors when compared with the contralateral side that imposed a lower force (51). Furthermore, the increment attained from impulse during a CMJ strongly correlated to an increase in hip and lumbar bone mass accretion (52), demonstrating that impulse has been associated to bone characteristics in research.

Force plate metrics have been used during a variety of exercise interventions to monitor load and the effects it has on bone. However, the validity and reliability of them to determine bone load has not been shown in applied sporting environments. The logistical challenges of this method mean that it cannot be implemented in day-to-day training for most sports, but, based upon the current literature, those who can do this should consider using force plates for monitoring adaptations to bone as higher GRF has been associated to greater bone accrual (53).

### IMU and bone

IMU's were the second most used method to estimate bone load with impact load, step count and PPA (Table 2). IMU's are small, moveable devices that provide a large amount of data to collect, process, analyse and report (54). They offer a viable option as they can be used in an applied setting, due to their small size and light weight, and can be mounted on specific anatomical locations to measure segmental external loading. This allows segmental information, such as the tibia, to be gathered, which is not the case with GPS or force plates. Segmental accelerations are shown to have a weak relationship with centre of mass accelerations (55), which highlights the importance of knowing what it is the user is wanting to measure so the correct measurement tool can be chosen.

IMU's have been shown to reliably monitor impact load, step count, and step intensity during dynamic team sport tasks (22), although no studies have investigated IMU metrics in relation to bone. Bone specific metrics have been developed within IMU devices (56). A surrogate measure of bone stress labelled “bone stimulus” represents the cumulative nature of impacts and predict the mechanical stimuli responsible for bone remodelling (22), however there is no published evidence of the IMU metrics correlating with actual changes in bone.

Using IMUs on anatomical positions unspecific to the area of interest may lead to less accurate results for the movements being performed (57). Therefore, it is essential that the location of the IMU devices are accurate when using loading metrics to understand the effect sport specific tasks have on bone. Overall, a small amount (17%) of support staff use IMUs, however the prevalence of those that do use IMUs to estimate bone load is high (Table 2), which shows the applicability of the method. As further research is undertaken, the relevance of IMU devices for monitoring external load in the field, and their association to bone characteristics will become clearer.

### Motion capture and bone

The application of motion capture, particularly 3D analysis techniques, can create predictive models of movement patterns that may reduce injury likelihood (58). Unfortunately, motion capture is not widely employed to monitor external load by support staff, although we have shown that when it is used, the prevalence of use in relation to bone is relatively high (Table 2). In theory, this technique can offer the greatest insight into the load being applied to bone due to its ability to create internal models of the musculoskeletal system, but the application is limited in most sporting environments as this method is time consuming and does not provide real time feedback.

The metrics Torque (*n* = 2; 9.1%), Moments (*n* = 2; 9.1%) and Stiffness (*n* = 3; 13.6%) were used by support staff to monitor external load. Higher knee joint stiffness can create higher loading rates that have been strongly associated with the estimation of bone load (19, 38, 59), which may be why support staff use stiffness as an informative metric. Kinetic data significantly correlates with BMD in elderly women, but not men, during walking and hip power-time and maximal hip power can predict 25.4% of femoral neck BMD (60). This finding was supported by a decrease in hip power correlating with a decrease in BMD in postmenopausal women (61). However, these studies only assessed habitual walking, and therefore it could be suggested that dynamic, high intensity sporting movements may offer additional increases in BMD at specific locations since running is likely to result in greater mechanical loading compared to walking (62). Comparatively, these increases are shown to be 2-9% higher in bone compression and tension, and 10-26% higher in shear stress, when running compared to walking (62). This increase can be used to harness a positive adaptation in bone and minimise injury risk by creating training programmes that expose athletes to gradual increases in load. Therefore, the findings (60, 61) suggest metrics derived from motion capture may offer informative data relative to changes in bone and the beneficial effect loading can have on special populations.

The use of motion capture by support staff to inform themselves on bone related outcomes is low. This is likely due to the time-consuming nature and expertise required for both data collection and data analysis. For these reasons motion capture techniques are mainly used by researchers as opposed to support staff. However, the development of marker less motion capture may widen the opportunity to integrate motion capture systems into applied environments.

### Limitations

The responses collected offer a general consensus for support staff working alongside athletes in an applied setting. The support staff were responsible for a large number of athletes (1,000+) across 5 continents with the majority (85%) competing at a national/international level therefore the authors are confident the sample is representative of support staff working in an elite environment. Recall bias may have impacted the answers supplied by the support staff. As those who completed the survey were all currently active at their organisations, however, the recall bias should be minimal, given the fact that they would have been largely referring to current practices. This study was exploratory in attempting to understand how external load is monitored in relation to bone. As such, the survey tool used was not validated, although this is offset by the novelty of the research approach.

### Practical applications

Despite the commonality of BSI being acknowledged (71%) by support staff in the survey, few support staff monitor external load to estimate bone load. Excessive loading is known to contribute to BSI, therefore monitoring and acting upon an athletes external load to estimate bone load may help reduce the burden of BSI. This study shows the number of support staff monitoring external load, what equipment/metrics they use and if they use those metrics to estimate bone load. There are a variety of methods that are available to support staff to inform on bone characteristics in their environments, however each of these methods are acknowledged to have their limitations which must be considered by support staff before implementing the methods.

## Conclusion

The findings show external load monitoring is commonly used in sporting environments but is seldom used to estimate bone load. There is no consistent measurement tool or metric that is invariably applied in relation to bone by support staff or within research. GPS is the most commonly used method to estimate bone load by support staff, but there is a lack of research associating GPS metrics with bone load. Accelerometry based data were shown to be the most prevalent method in relation to bone, but lacked generic popularity amongst support staff. Force plates were reported to be the second most popular method for monitoring external load (48%), but the least prevalent to estimate bone load. The application of the methods to estimate bone load within applied environments is challenging due to the cost, time and expertise of the methods. Research exploring the most applied methods for monitoring external load to estimate bone load in applied environments are recommended.

## Data Availability

The raw data supporting the conclusions of this article will be made available by the authors, without undue reservation.
