# Supplementary material for: Perspectives from research and practice: A survey on external load monitoring and bone in sport
Source: Front Sports Act Living. 2023 Apr 25;5:1150052. doi: 10.3389/fspor.2023.1150052 (PMC10166824; doi:10.3389/fspor.2023.1150052)
Supplement: Supplementary file 1 [file Datasheet1.pdf]

External load monitoring in the field: Online questionnaire

Participant Information Sheet

External load can be defined as the work completed by an athlete independent of their internal characteristics i.e., acceleration, force, etc. This is not to be confused with internal load which is the biological stresses imposed on an individual (Bourdon et al., 2017) i.e., heart rate, blood lactate, etc. External load monitoring in an applied setting is an area of interest in research due to its association to injury and performance (Halson, 2014). We are interested in finding how current practitioners monitor external load within their field and whether they relate this load to bone in any way.

This questionnaire is designed to gather information on athlete external load monitoring practices within an applied sport and exercise environment. Insight into the monitoring methods used in elite sport will provide a more unified and accurate understanding of how external load is monitored. To take part you should be over 18 and be employed in the field of sport and exercise. You are relevant to this study no matter what level of sport you work in (amateur or professional).

Completion of this questionnaire will take approximately 5-10 minutes. Your participation is voluntary, all the data that you provide will remain anonymous and you will not be identifiable in the dissemination of this research. Your data will be stored securely on a security protected hard drive. If you wish to withdraw your consent after data collection, you have up to two weeks after completion to do so. You can do this by contacting the researchers via email, details supplied at the end of the questionnaire. If you have any questions prior to completing the questionnaire, please contact the researcher below before commencing.

Contact

Researcher
Reece Scott: reece.scott@ntu.ac.uk

Supervisor
Dr Ian Varley: ian.varley@ntu.ac.uk

Consent form

If you agree to participate in the project outlined above, please read the following statements before continuing to the questionnaire.

I understand how to complete the questionnaire and agree to do so as honestly as I can.

I have had the opportunity to ask any questions, communicate and discuss any concerns and queries associated with the study.

I understand that my participation is voluntary, and I have the right to withdraw or discontinue participation at any time with no obligation to provide reasons behind the decision.

I understand that my responses in the questionnaire will be recorded and analysed for content.

I understand that all information which I have provided will be treated as private and confidential and communicated to others with my identity concealed.

I understand that I can withdraw my data using my unique identifier after I've completed the survey up until 2 weeks after completing the survey.

I confirm I am aged 18 or over.

I can confirm that I meet the participant criteria.

- Yes
- No

Please create a unique identifier code; this should be a random 5 letters and/or numbers which you will need to keep in case you wish to withdraw from the study i.e., DI8HV. **Please make a note of your ID code for your records. Please do not include numbers/letters which we may link you to i.e., Name of your club.**

Demographics

The following questions are necessary to the research and will be used to create an anonymous profile of you, as a practitioner. This information will not be used to identify you or reveal your identity.

1. Which sport(s) do you work in? *Select all that are applicable.*

- Football/Soccer
- Cricket
- Basketball
- Hockey
- Tennis
- Volleyball
- Rugby
- American football
- Baseball
- Athletics
- Other (please specify): ___________________________________

2. What gender do you **primarily** work with? *Please select one.*

- Male
- Female
- Other

3. Which Continent do you work in? *Please select one.*

- Africa
- Asia
- Australia

- Europe – UK
- Europe – Rest of Europe
- North America
- South America

4. What role are you primarily employed as within the sport? *Please select one.*

- Physiotherapist
- Strength and Conditioning
- Sport Scientist
- Doctor
- Coach
- Other (please specify): ___________________________________

5. What age group of athletes do you work with? *Select all that are applicable.*

- Under 16
- 16 - 18
- 19 - 21
- 22 +

6. What level of sport do you currently work in? *Please select one.*

- International
- National
- University/Collegiate
- Regional
- Other (please specify): ________________________

External Load

External load can be defined as the **work completed by an athlete independent of their internal characteristics i.e., acceleration, force, etc.** This is not to be confused with internal load which is the biological stresses imposed on an individual (Bourdon et al., 2017) i.e., heart rate, blood lactate, etc.

7. Does your club/organisation monitor external load in your athletes?

- Yes
- No

If NO, goes to question 16.

Load Monitoring

8. How do you primarily use external load data? *Select all that are applicable.*

- Monitor Rehabilitation
- Inform on susceptibility to injury

- Inform performance
- Track performance
- Monitor the effectiveness of a training programme
- Other (please specify): _______________________________

9. What systems do you use to monitor external load? *Select all that are applicable.*

- Global Positioning System (GPS)
- Inertial Measuring Unit (IMU)
- Motion analysis
- Force plates
- Surface Electromyography (sEMG)
- Other (please specify): _____________________________

10. What are the main outputs you use? *Select all that are applicable.*

- GPS – PlayerLoad
- GPS – Total distance covered
- GPS – High speed distance
- IMU – Impact Load
- IMU – Step Count
- IMU – Peak positive acceleration
- Motion Analysis – Torque
- Motion Analysis – Moment
- Motion Analysis – Stiffness
- Force Plates – Peak ground reaction force
- Force Plates – Rate of force development
- Force Plates – Impulse
- sEMG – Amplitude
- Other (please specify): _____________________________

11. When do you monitor external load? *Please select one.*

- Continuously (during training/competition)
- Intermittently (for testing purposes)
- When recovering from injury
- Other (please specify): ________________________

12. Have overuse stress related bone injuries occurred within your club/organisation?

- Yes
- No
- Unsure

13. Do you use any of the external load metrics attained to estimate load on bone?

- Yes
- No

- Unsure

If NO, go to question 17.

Bone Monitoring

By **bone monitoring we are referring to using external load methods and relating them to bone in any form**. It is important to know whether these methods have been translated into applied settings to understand if they are replicable and relevant to practitioners.

14. What are the **main outputs** you use specifically in relation to bone? *Select all that are applicable.*

- GPS – PlayerLoad
- GPS – Total distance covered
- GPS – High speed distance
- IMU – Bone Stimulus
- IMU – Impact Load
- IMU – Step Count
- Motion Analysis – Torque
- Motion Analysis – Moment
- Motion Analysis – Stiffness
- Force Plates – Peak ground reaction force
- Force Plates – Load rate
- Force Plates – Impulse
- sEMG – Amplitude
- Other (please specify): ______________________________

15. When do you collect data? *Please select one.*

- Training
- Competition
- Training and Competition
- Other (please specify): ________________________________

16. What is the primary reason you don't monitor external load?

- Lack of time
- Lack of equipment
- Lack of knowledge
- Don't feel it is needed
- Other (please specify): ________________________________

Only answered as result of NO from Question 7.

17. What is the primary reason you don't relate external load to bone?

- Lack of time

- Lack of equipment
- Lack of Knowledge
- Don't feel it is needed
- Don't believe external load can be related to bone
- Other (please specify): ______________________________

Only answered as result of NO from Question 13.

Sorry, you are unable to complete this questionnaire due to not meeting the minimum criteria (age and consent).

Only shown as result of NO from Consent form.

Final Page – Thank you

Thank you for taking the time to complete this questionnaire. If you have any questions regarding your participation in this research or wish to withdraw your data, please contact the research team.

If you are interested in the current studies related to external load and bone at NTU then please email the researcher below with your details.

Researcher
Reece Scott: reece.scott@ntu.ac.uk

Supervisor
Dr Ian Varley: ian.varley@ntu.ac.uk
